# Evaluating Scholars’ Impact and Influence: Cross-sectional Study of the Correlation Between a Novel Social Media–Based Score and an Author-Level Citation Metric

**DOI:** 10.2196/28859

**Published:** 2021-05-31

**Authors:** Lucas Oliveira J e Silva, Graciela Maldonado, Tara Brigham, Aidan F Mullan, Audun Utengen, Daniel Cabrera

**Affiliations:** 1 Department of Emergency Medicine Mayo Clinic Rochester Rochester, MN United States; 2 Mayo Clinic Libraries Mayo Clinic Florida Jacksonville, FL United States; 3 Department of Biostatistics and Informatics Mayo Clinic Rochester Rochester, MN United States; 4 Symplur Los Angeles, CA United States

**Keywords:** social media, Twitter, journal impact factor, h-index, digital scholarship, digital platform, Scopus, metrics, scientometrics, altmetrics, metrics, stakeholders, health care, digital health care

## Abstract

**Background:**

The development of an author-level complementary metric could play a role in the process of academic promotion through objective evaluation of scholars’ influence and impact.

**Objective:**

The objective of this study was to evaluate the correlation between the Healthcare Social Graph (HSG) score, a novel social media influence and impact metric, and the h-index, a traditional author-level metric.

**Methods:**

This was a cross-sectional study of health care stakeholders with a social media presence randomly sampled from the Symplur database in May 2020. We performed stratified random sampling to obtain a representative sample with all strata of HSG scores. We manually queried the h-index in two reference-based databases (Scopus and Google Scholar). Continuous features (HSG score and h-index) from the included profiles were summarized as the median and IQR. We calculated the Spearman correlation coefficients (ρ) to evaluate the correlation between the HSG scores and h-indexes obtained from Google Scholar and Scopus.

**Results:**

A total of 286 (31.2%) of the 917 stakeholders had a Google Scholar h-index available. The median HSG score for these profiles was 61.1 (IQR 48.2), and the median h-index was 14.5 (IQR 26.0). For the 286 subjects with the HSG score and Google Scholar h-index available, the Spearman correlation coefficient ρ was 0.1979 (*P*<.001), indicating a weak positive correlation between these two metrics. A total of 715 (78%) of 917 stakeholders had a Scopus h-index available. The median HSG score for these profiles was 57.6 (IQR 46.4), and the median h-index was 7 (IQR 16). For the 715 subjects with the HSG score and Scopus h-index available, ρ was 0.2173 (*P*<.001), also indicating a weak positive correlation.

**Conclusions:**

We found a weak positive correlation between a novel author-level complementary metric and the h-index. More than a chiasm between traditional citation metrics and novel social media–based metrics, our findings point toward a bridge between the two domains.

## Introduction

Since the development of social media platforms and new communication channels, the use of traditional bibliographic metrics (ie, citation counts, h-indexes) as the predominant factors for academic performance has been questioned [1]. Traditional benchmarks such as citation counts fail to capture the authors’ impact outside academic circles [2]. The ways in which research output is indexed, searched, located, read, and mentioned have significantly changed, and these ways do not describe the influence and impact that scholarly work may have outside core academic domains [3,4].

In the health care world, social media platforms (eg, Twitter, Facebook) are consistently used by patients, policy makers, clinicians, and researchers as efficient ways of sharing information, staying up to date with scientific knowledge, and collaborating with peers and patients [5]. The widespread use of social media by health care stakeholders has led to the development of alternative impact metrics, also known as “altmetrics” [6]. The altmetrics approach offers new ways to analyze and inform scholarship [7]. It complements rather than replaces traditional indicators of a scholar’s performance [8]. Altmetrics have even been adopted aggressively by traditional publishing companies [9]. The study of these alternative metrics is an emerging field; unlike traditional parameters, such as the impact factor or h-index, it does not rely solely on citation counts but is a composite measure. It considers other features such as the number of knowledge databases that refer to the work, and the number of times the work has been viewed and downloaded; it also factors in the number of mentions in social media and traditional news outputs.

Academic merit and achievement should be appraised using frameworks such as the comprehensive researcher achievement model (CRAM) [8], encompassing a combination of four aspects: quantity of researcher outputs (productivity), value of outputs (quality), outcomes of research outputs (impact), and relations between publications or authors and the wider world (influence). Current traditional benchmarks focus mostly on productivity and quality, while alternative metrics focus on influence and impact. In 2011, Eysenbach proposed the Twimpact Factor, an article level social media impact metric consisting of the absolute cumulative number of tweetations 7 days after publication of the article, and the Twindex, which is the relative percentile of the Twimpact Factor of a given article compared with other articles in the same journal [10]. For articles published in the *Journal of Medical Internet Research*, Eysenbach found relatively strong article-level correlations between these metrics (number of tweets, adjusted by time and journal factors) and future citations and highlighted the importance of using social media–based impact measures to complement traditional citation metrics [10]. While social media metrics at the article or journal level already exist and have been correlated with traditional citation metrics [10], novel tools could also be used to evaluate features such as influence and impact at the author level. There is a clear need to improve the ways in which the different outputs of scholarly work are evaluated, as claimed by the Declaration on Research Assessment (DORA) movement [11]. The development of an author-level complementary metric could play a role in the academic promotion process through objective evaluations of scholars’ influence and impact.

Recently, multiple organizations have created tools that attempt to measure influence and impact in the digital domain primarily by using network analysis of social media activity and digital publications [12]. Among these innovations, Symplur’s Healthcare Social Graph (HSG) score has recently emerged [13]. In this context, we aimed to evaluate the correlation between the HSG, a social media influence and impact metric, and the h-index, a traditional author-level metric.

## Methods

### Study Design, Study Setting, and Participants

This report was written following the Strengthening the Reporting of Observational Studies in Epidemiology (STROBE) guidelines [14]. This study was deemed exempt by the Institutional Review Board.

This was a cross-sectional observational study of health care stakeholders with a social media presence randomly sampled from the HSG database in May 2020. Health care stakeholders included the following three taxonomic categories: “doctor” (ie, those identified as possibly licensed, MDs, DOs, PhDs), “health care professionals” (ie, those identified as other health care professionals such as nurses, dietitians, respiratory therapists, and pharmacists), and “researchers/academicians” (ie, people working in the field of health-related research or academia). Over 1 million Twitter profiles were labeled according to the health care stakeholder category as part of the database metadata. Only the profiles of those identifying themselves in their public Twitter profile received a label by Symplur partly through manual verification and partly through a machine learning process [15]. We did not exclude health care stakeholders based on their discipline. Considering the 6 million Twitter accounts that received an HSG score and individuals identified as health care stakeholders in May 2020, we performed stratified random sampling to obtain a representative sample with all strata of HSG scores. A random sample of 100 profiles from each HSG score decile (0-9, 10-19, etc) was obtained, yielding an initial list of 1000 subjects with their respective HSG scores. This stratification method was chosen owing to the skewness of the HSG scores in the Symplur database, where simple probability sampling would lead to a study population restricted to lower values of the HSG score.

### Data Source, Variables, and Measurement

Symplur is a health care social media analytics company that created the HSG database holding public digital content (ie, conversations, interactions) originating from Twitter and obtained via the official Twitter application programming interface (API) while supplementing it with other public content from social media platforms including LinkedIn, YouTube, Instagram, Reddit, and Facebook. The HSG score was developed by Symplur to identify and rank influencers in any health care topic and is conceptually like an eigenvector [16]. This score ranks Twitter accounts by their global conversational impact in healthcare over the last 52 weeks. As long as Twitter accounts have engaged (ie, tweeted at least once) in one of the 40,000 health care terms being tracked, they will be evaluated. The score is not determined by the absolute numbers of tweets or how many mentions they have received for the given time period, but by the impact of the posted messages. The score comprises three components, a social network analysis algorithm, health care stakeholder weighting, and conversation quality algorithm. The network analysis algorithm is inspired by the hyperlink-induced topic search (HITS) algorithm [17] and considers each Twitter account’s conversation graph by recursively analyzing the health care influence of each individual conversation partner, the influence of the conversation partner’s own conversation partners, and so on [18]. In this respect, it is similar to modern impact factor algorithms for academic journals and Google’s PageRank [19]. The score is designed specifically for health care and considers the health care stakeholder groups to which the account holders belong. In other words, it matters what role a person has in health care. If, for example, an account is interacting with or being mentioned by another account that is not related to health care, then those conversations and mentions will have less weight as determined by the algorithm. If, on the other hand, the conversations and mentions are made by a health care stakeholder, then that has more weight according to the algorithm. Based on the analysis of these conversations, a quality score is factored with a conversation volume to provide a weighted measure for the impact scores. After that, the 52 weekly rankings and quality scores are combined into a single number for each social media profile and then normalized on a scale of 0 (very low influence) to 100 (very high influence).

For each of the 1000 Twitter profiles initially included in our stratified random sample, we manually queried their h-indexes in two reference-based databases (Scopus and Google Scholar) by searching their names in each profile service’s search engine. Before extracting the data, we used a standardized verification process to confirm if the identified profiles corresponded to the Twitter user. Any profile found in Google Scholar or Scopus was verified using at least three of the following identifiers: name (first, middle, last), title, location (country/city), field/specialty, affiliation, and qualitative analysis of Twitter conversations or a free-form Google search using the associated name and any other identifier available. Once a profile was found in either of the platforms and the verification process confirmed with at least three identifiers, the h-index was extracted. This verification process was created to decrease the probability of extracting data from an incorrect profile (eg, similar name but not the author of interest). In Google Scholar and Scopus, the h-index is calculated as “number *n* of a researcher's papers that have all received at least *n* citations” [20]. Although there have been multiple studies [20-24] that have highlighted the advantages, disadvantages, and variations, no other traditional author-level metric has had the same level of acceptance or resilience over the past 15 years. Individuals can calculate the h-index of any researcher as long as they have access to a resource providing the citation count of that researcher's publications or research objects. The three most prominent resources or platforms that provide citation counts for researchers are the Web of Science (Clarivate Analytics – previously of Thomson Reuters), Scopus (Elsevier), and Google Scholar (Alphabet). To provide a more comprehensive reporting for this study, Scopus and Google Scholar were chosen to provide the traditional/benchmark h-index data for each individual. This decision was based on the 2018 study by Martin-Martin et al [25], which found that the greatest inclusion of citations in Health & Medical Sciences was on Scopus and Google Scholar. Additional factors were considered when choosing between Web of Science and Scopus. Scopus was seen as providing all authors better access to their own author profiles, which would allow authors to clarify their publications and correct inaccuracies. Additionally, a 2016 study by Walker et al [26] showed a higher interrater reliability in Scopus than in Web of Science for the h-index calculation.

The h-index for the first 100 profiles was independently extracted by three independent investigators (LOJS, GM, and TB). In this initial set of 100 profiles, there was a 98% overall agreement for the h-index extracted from Google Scholar and a 96% overall agreement for the h-index extracted from Scopus. Disagreements were discussed and resolved through consensus with the senior author (DC). Once our standardized verification process and data extraction methods exhibited adequate reliability, the remaining profiles were extracted independently; 600 were reviewed by the first author (LOJS) and 150 by each of the two other investigators (GM, TB). Investigators extracting the h-index for these profiles were blinded to the HSG scores of all subjects.

### Data Analysis

From the initial list of 1000 subjects, we excluded those with incomplete names or non-individual user profiles. The remaining profiles were included for the main data analysis if an h-index was available from either Google Scholar or Scopus. All analyses were conducted using the BlueSky Statistics (Version 7.0.746.34007) graphic user interface (GUI) for R. Continuous features (HSG score and h-index) from included profiles were summarized as the median and IQR. Correlation analyses were performed between the HSG scores and h-index obtained from Google Scholar (overall h-index and 2015 h-index) and Scopus (overall h-index). Given the highly skewed nature of metrics such as the h-index [27], we calculated the Spearman correlation coefficient (ρ). This is similar to the Pearson correlation, but it is based on ranks rather than original values. Like the Pearson correlation, values range from –1 to +1, with larger absolute values indicating a stronger relationship. A correlation *t* test was conducted to evaluate the statistical significance of the correlation coefficients. *P* values <.05 were considered statistically significant. For sensitivity analysis, we considered the h-index as 0 for those subjects in which a Scopus h-index was not found.

Simple linear regression was initially implemented to understand the linear relationship between the HSG score and h-index (Google Scholar and Scopus). To better understand the true relationship between the HSG score, and the overall h-index provided by Google Scholar and Scopus, negative binomial hurdle regression was performed. The h-index was used as the response variable, and the HSG score was the predictor of interest. A negative binomial model was chosen owing to the skewed nature of the h-index data and the overdispersion present in the data distribution. To account for the high number of zeroes not covered by a negative binomial distribution, a hurdle model with a binomial logistic link function was also implemented. Model selection was performed using the Vuong test and the Akaike information criteria (AIC).

## Results

### Twitter Profiles

Our stratified random sample generated an initial list of 1000 Twitter profiles from the Symplur database. Of these, 83 were excluded for the following reasons: 5 were repeated profiles, 62 had incomplete names on Twitter (ie, no first and last names, making it impossible to search for a corresponding Google Scholar or Scopus profile), and 16 were not individual user profiles. Among the 917 individual Twitter profiles with complete names for which h-indexes were searchable, 429 (46.8%) were from the United States, 173 (18.9%) from the United Kingdom, 54 (5.9%) from Canada, 49 (5.3%) from Spain, 41 (4.5%) from Australia, 17 (1.9%) from India, 13 (1.4%) from the Netherlands, 13 (1.4%) from France, 12 (1.3%) from Ireland, 9 (1.0%) from Brazil, and the remaining 11.6% from 36 other countries from all continents (only 5 profiles were from unknown countries).

A total of 286 (31.2%) of the 917 stakeholders had a Google Scholar h-index available. The median HSG score for these profiles was 61.1 (IQR 48.2), and the median h-index was 14.5 (IQR 26). A total of 715 (78%) of the 917 stakeholders had a Scopus h-index available. The median HSG score for these profiles was 57.6 (IQR 46.4), and the median h-index was 7 (IQR 16).

### Google Scholar h-Index

**Figure 1:**
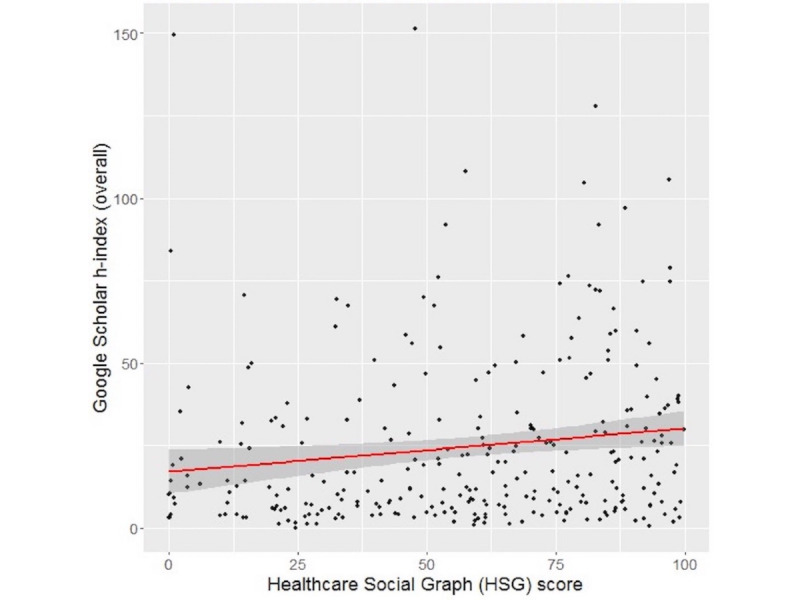
Correlation between HSG scores and Google Scholar overall h-indexes. Spearman correlation coefficient ρ=0.1979 (N=286). The red line is the regression line; the shaded area is the 95% CI.

**Figure 2:**
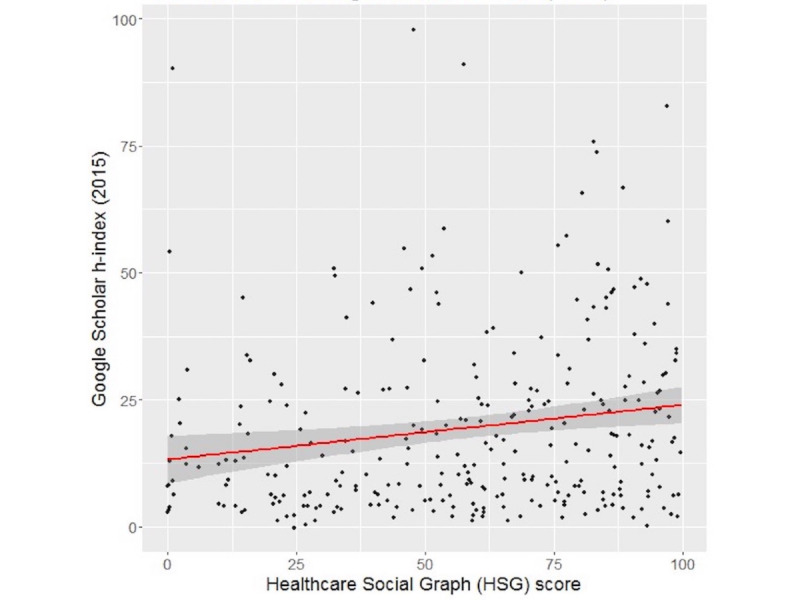
Correlation between HSG scores and Google Scholar 2015 h-indexes. Spearman correlation coefficient ρ=0.203 (N=286). The red line is the regression line; the shaded area is the 95% CI.

For the 286 subjects with the HSG score and overall h-index provided by Google Scholar available, the Spearman correlation coefficient ρ was 0.1979 (*P*<.001), indicating a weak positive correlation between these two metrics (Figure 1). When we analyzed the correlation for the 2015 h-index from Google Scholar, the results were similar (ρ=0.203) (Figure 2). Also, when we analyzed the Google Scholar i10 index, the results did not change significantly (see Multimedia Appendix 1 and Multimedia Appendix 2).

Linear regression using the Google Scholar overall h-index as the response found a significant association between the HSG score and h-index. Assuming a linear relationship, for every 10-point increase in the HSG score, there was an associated increase of 1.134 in the h-index (95% CI 0.280-2.347; *P*=.01). The *R^2^* value was 0.0214 and the linear regression equation is expressed as (E[Google Scholar overall h-index] = 17.037 + 0.1314*[HSG score]). From the negative binominal hurdle model, there was no effect of the HSG score on whether an author’s Google Scholar h-index is 0 or positive (log-odds=0.043; *P*=.31). However, for authors with a positive h-index, a 5-point increase in the HSG score was associated with a 2.7% increase in Google Scholar h-index (exp[coef]=1.027; 95% CI 1.006 -1.048; *P*=.01). Additionally, the Vuong test found that the hurdle model was a better fit than the negative binomial model (*z* statistic=3.092; *P*<.001).

### Scopus h-Index

**Figure 3:**
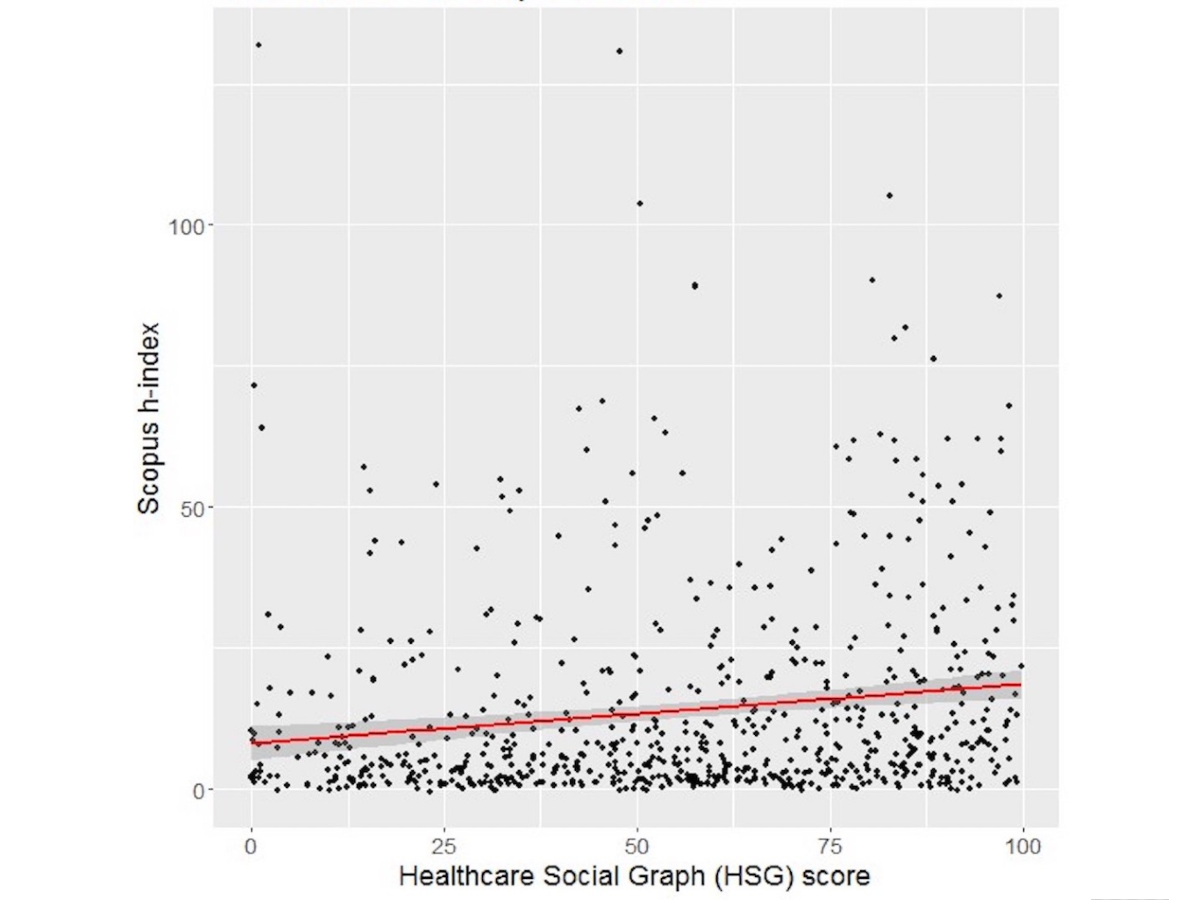
Correlation between HSG scores and Scopus h-indexes. Spearman correlation coefficient ρ=0.2173 (N=715). The red line is the regression line; the shaded area is the 95% CI.

For the 715 subjects with the HSG score and Scopus h-index available, the Spearman correlation coefficient ρ was 0.2173 (*P*<.001), also indicating a weak positive correlation (Figure 3). In the sensitivity analysis, in which subjects without a Scopus h-index available were computed as having an h-index of 0, therefore including all 917 initially eligible profiles, the Spearman correlation coefficient ρ was 0.317 (*P*<.001) (see Multimedia Appendix 3).

Univariate linear regression fitting of Scopus h-indexes found a significant association with the HSG score. Assuming a linear relationship, for every 10-point increase in the HSG, we expect a 1.049-point increase in the h-index (95% CI 0.567-1.530; *P*<.001). The *R*^2^ value was 0.0249 and the linear regression equation is expressed as (E[Scopus h-index] = 8.0821 + 0.1048*[HSG score]). From the negative binomial hurdle model, we found no significant effect of the HSG score on whether an author’s h-index is 0 or positive (log-odds=0.0072; *P*=.27). However, for authors with a positive h-index, a 5-point increase in the HSG score was associated with a 4% increase in h-index (exp[coef]=1.040; 95% CI 1.021-1.061; *P*<.001). The Vuong test found that the hurdle model was a better fit than the negative binomial model (*z* statistic=4.606; *P*<.001).

## Discussion

### Principal Results

The advent of digital scholarship is rapidly changing the way scholarship is created and appraised in academia. We are currently seeing a swift transition from a paradigm in which the impact of an academician was circumscribed to deliverables critiqued by a restrictive circle of peers to a novel model in which the importance of scholarly work is measured by the influence and impact it generates in academic circles and among the general public. This leads to the critical need to adopt new appraisal concepts and tools [1,10,28].

The HSG score represents a novel author-level tool within the domain of altmetrics. This metric aims to measure and illustrate the influence a particular stakeholder has in health care social media as a function of user-generated content and interactions. This method is common for analyzing the weight or importance of specific users that are part of larger networks [29]. In general, the more the connections and the more information users create or are involved with, the greater their importance in a network [30].

Citation-focused metrics such as the h-index assess the importance of academicians based on the number of times their work has been cited by other scholars, with a significant bias constructed to value certain outputs (eg, prestigious journals) more than others. This distorts the organic reach and impact of articles (eg, where bad articles in good journals are valued more than good articles in bad journals), conflagrating production and publishability with influence and impact [31].

In this study, we aimed to find if there is a relationship between the HSG score, a marker for influence in a network, and the h-index, a metric for productivity. The assumption driving this comparison was that a high degree of productivity (greater h-index) would be associated with higher impact among stakeholders in the field and subsequent influence on digital networks.

Approximately three quarters of health care stakeholders identified by HSG scores had a concomitant h-index profile; this simple observation illustrates that there is a significant overlap between academic endeavors and the participation of these users in social media. In other words, academicians are part of general public forums such as social media; they value such forums and participate in them.

When analyzing the relation between the HSG score and h-index, we found a correlation, albeit a weak one, between the two metrics. This positive relation seems to indicate that the higher the HSG score, the higher the h-index (and vice versa). We believe the association describes a relation between scholarly productivity and influence in a health care network; this is possibly explained by academicians using digital domains to disseminate their scholarly work and subsequently bring attention to it, by measuring interactions with other stakeholders and organically increasing their connections and weight in the network. Nevertheless, it is important to mention that the low *R^2^* value approximately at 2% implies that although we have a statistically significant correlation and are capturing similar trends, there is a sizable amount of variability that is not shared between these two metrics. This emphasizes the simple fact that these metrics measure *different* components of scholarly work and should be evaluated in an independent and complementary way.

The HSG score and h-index are metrics that are of interest for scholars and academic establishments. Per their definitions, these tools are aimed at different aspects of the CRAM framework [8], where the HSG score likely appraises impact and influence and h-index productivity and quality. Remarkably, from our analysis, we can describe an association bridging these four aspects; the influence and impact of a user in a health care–specific digital network are correlated with their academic productivity and quality. More than a chiasm between traditional citation metrics and novel social media–based metrics, our findings point toward a positive relation between the two domains.

### Limitations

There are several limitations that need to be acknowledged. First, the accuracy of the metrics that were obtained (which were subsequently used to compute correlations) depends on the validity of the data provided by each reference-based database. Some of these platforms (eg, Google Scholar) can easily be manipulated [32]. Scopus automatically calculates the h-indexes of authors without a profile in their database, which explains why there were higher numbers of profiles and h-indexes available in Scopus when compared to Google Scholar, in which individuals need to create active profiles. In Scopus, authors may have more than one profile and, for this reason, we have used an available tool in their platform to combine profiles from the same author to obtain the most accurate h-index for that author. Second, Scopus and Google Scholar data are dynamic because new citations are constantly being added to their databases. As we were unable to automatically retrieve the h-indexes from these databases on the same day, manual data extraction occurred over a four-month period. Therefore, authors may have had their h-indexes extracted with a time difference as long as 100 days, and this, although unlikely, could have influenced the accuracy of our analysis. We assumed that the h-index would be time invariant (while in fact it is not) during the period of data extraction. Nevertheless, the h-indexes should theoretically be less dynamic than citations alone, and it is unlikely to change by a large magnitude even after a 100-day period [33]. Third, we have not considered the ages of the authors, which might have an impact on the correlation measures given that more experienced authors may exhibit distinctive behavior compared to emerging authors. Fourth, Google Scholar seems to overestimate author-level metrics when compared to Scopus owing to inclusion of gray literature citations, among other reasons. However, we extracted the h-indexes from both databases, and the results did not change when using one h-index over another. In fact, the h-indexes from Google Scholar and Scopus were strongly correlated with each other (see Multimedia Appendix 4).

### Conclusions

It appears that novel author-level altmetrics based on network analysis in social media and digital publications have a positive association with traditional bibliometric benchmarks. This seems to indicate that not only can they coexist but can also supplement and augment each other’s domains. Academicians interested in a comprehensive appraisal of their academic work and preparing for advancement need to be deliberate about investing time and attention into both spheres of appraisal (traditional and altmetrics), as they are relevant, significant, and most importantly appear to move in the same direction and amplify each other.

## Appendix

Multimedia Appendix 1 Correlation between HSG scores and Google Scholar overall i10 indexes. Spearman correlation coefficient ρ=0.2037 (N=286).

Multimedia Appendix 2 Correlation between HSG scores and Google Scholar 2015 i10 indexes. Spearman correlation coefficient ρ=0.2087 (N=286).

Multimedia Appendix 3 Correlation between HSG scores and Scopus h-indexes after computing a Scopus h-index of 0 for subjects without Scopus h-indexes. Spearman correlation coefficient ρ=0.317 (N=917).

Multimedia Appendix 4 Strong correlation between Scopus and Google Scholar h-indexes.

## Abbreviations

AIC: Akaike information criteria
API: application programming interface
CRAM: comprehensive researcher achievement model
DORA: Declaration on Research Assessment
GUI: graphical user interface
HITS: hyperlink-induced topic search
HSG: Healthcare Social Graph
IQR: interquartile range
STROBE: Strengthening the Reporting of Observational Studies in Epidemiology
