# Supplementary figures and images for: Evaluating Scholars’ Impact and Influence: Cross-sectional Study of the Correlation Between a Novel Social Media–Based Score and an Author-Level Citation Metric
Source: J Med Internet Res. 2021 May 31;23(5):e28859. doi: 10.2196/28859 (PMC8204234; doi:10.2196/28859)

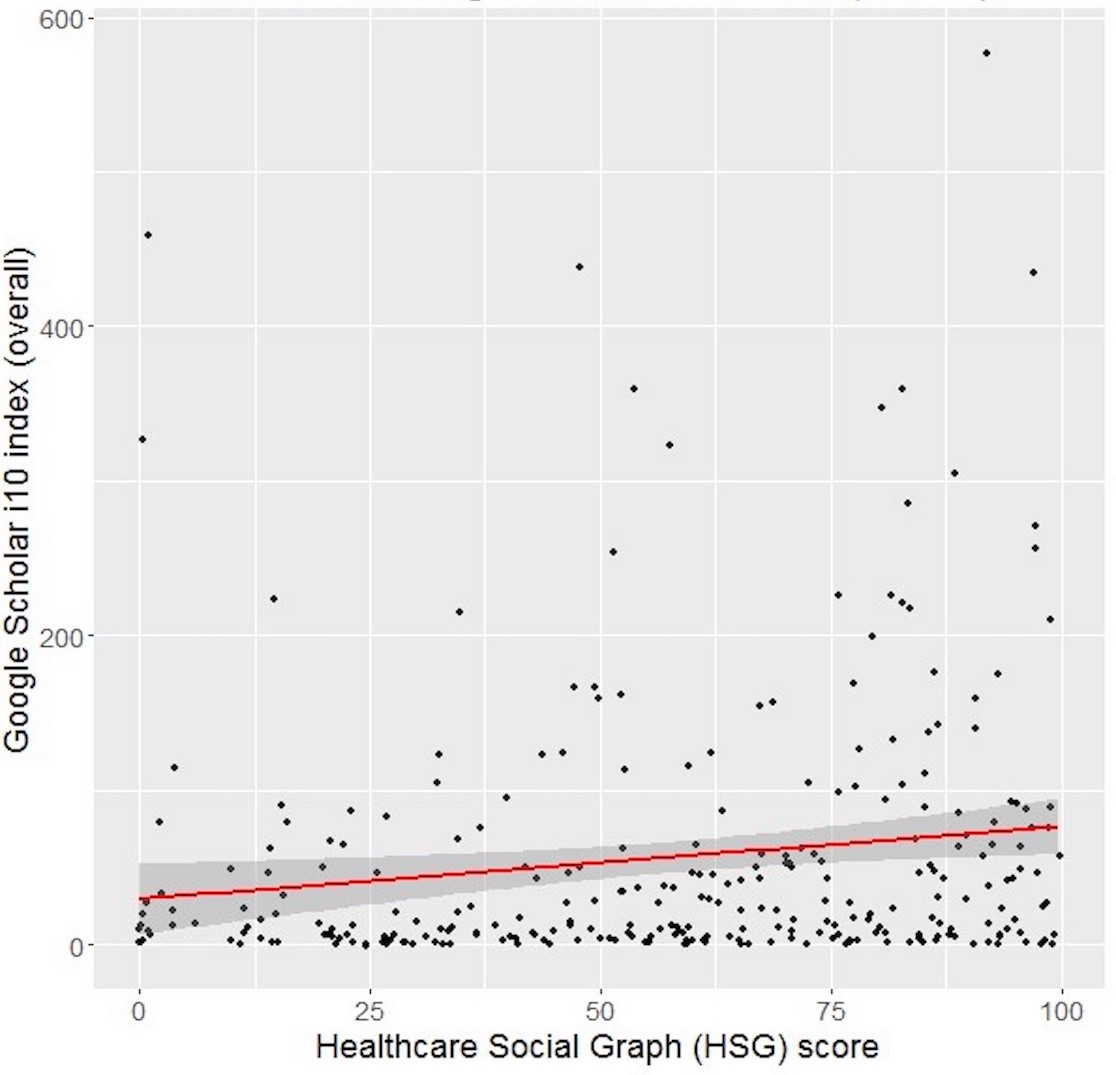

Supplement: Multimedia Appendix 1 [file jmir_v23i5e28859_app1.png]

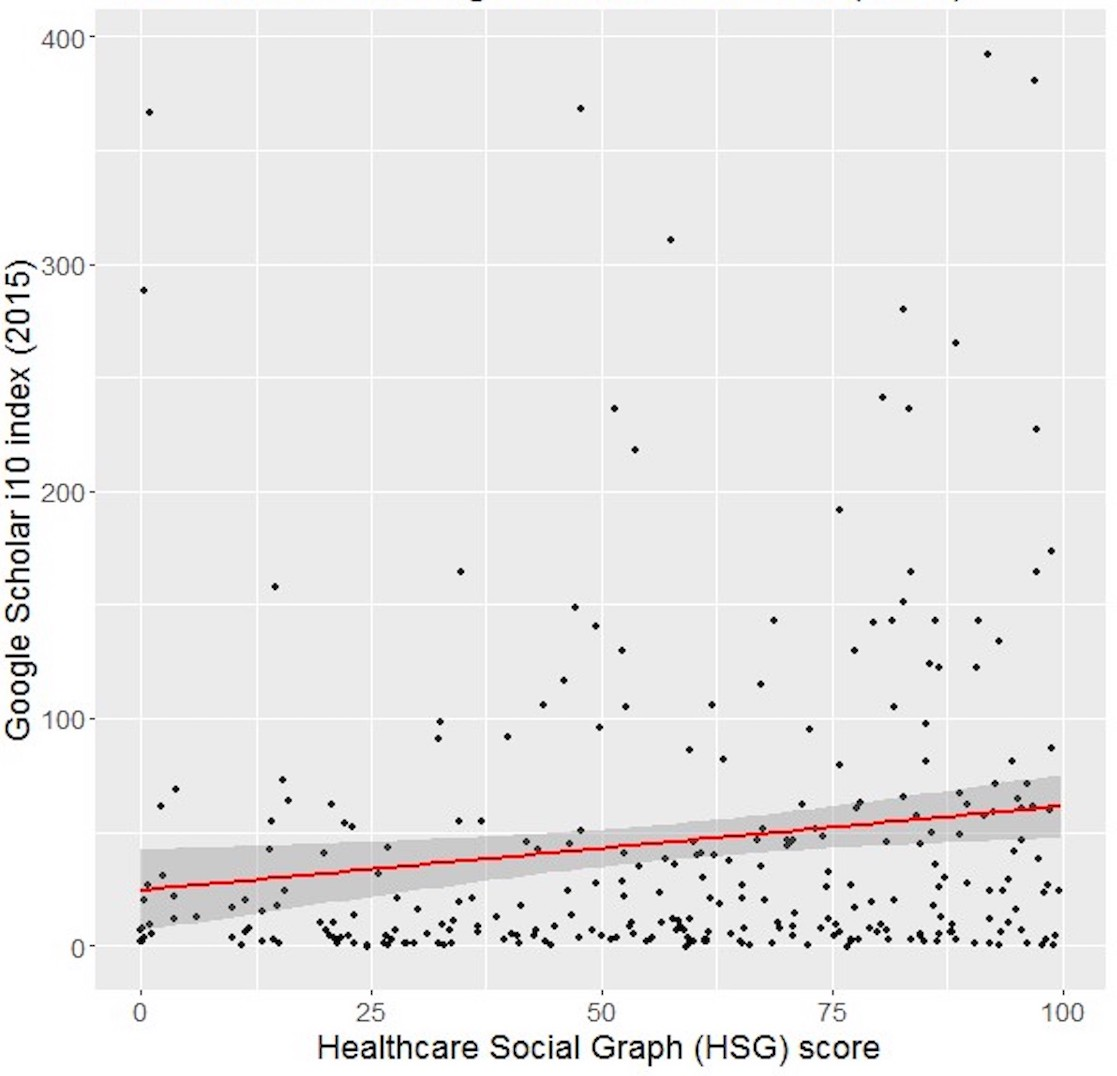

Supplement: Multimedia Appendix 2 [file jmir_v23i5e28859_app2.png]

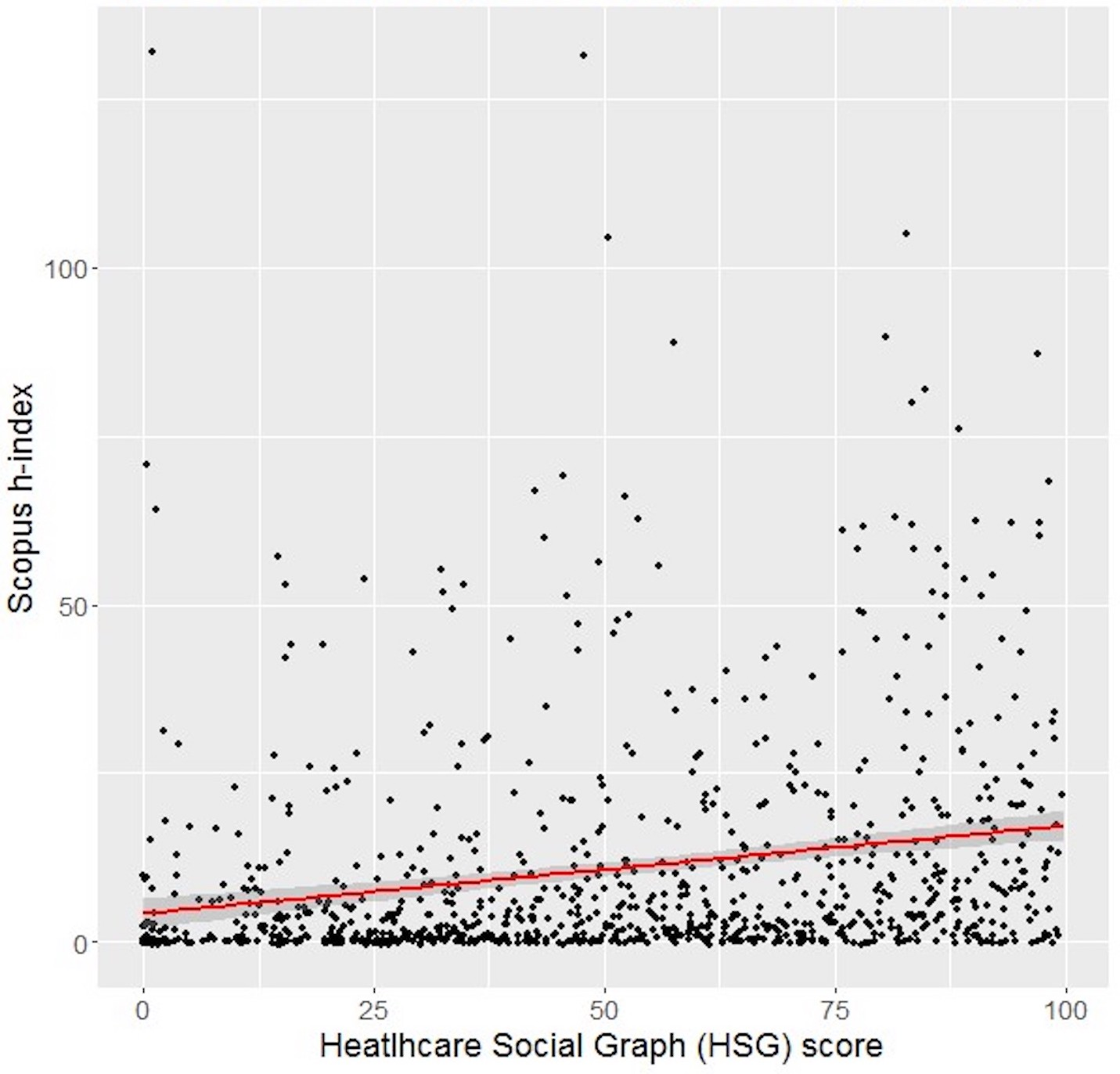

Supplement: Multimedia Appendix 3 [file jmir_v23i5e28859_app3.png]

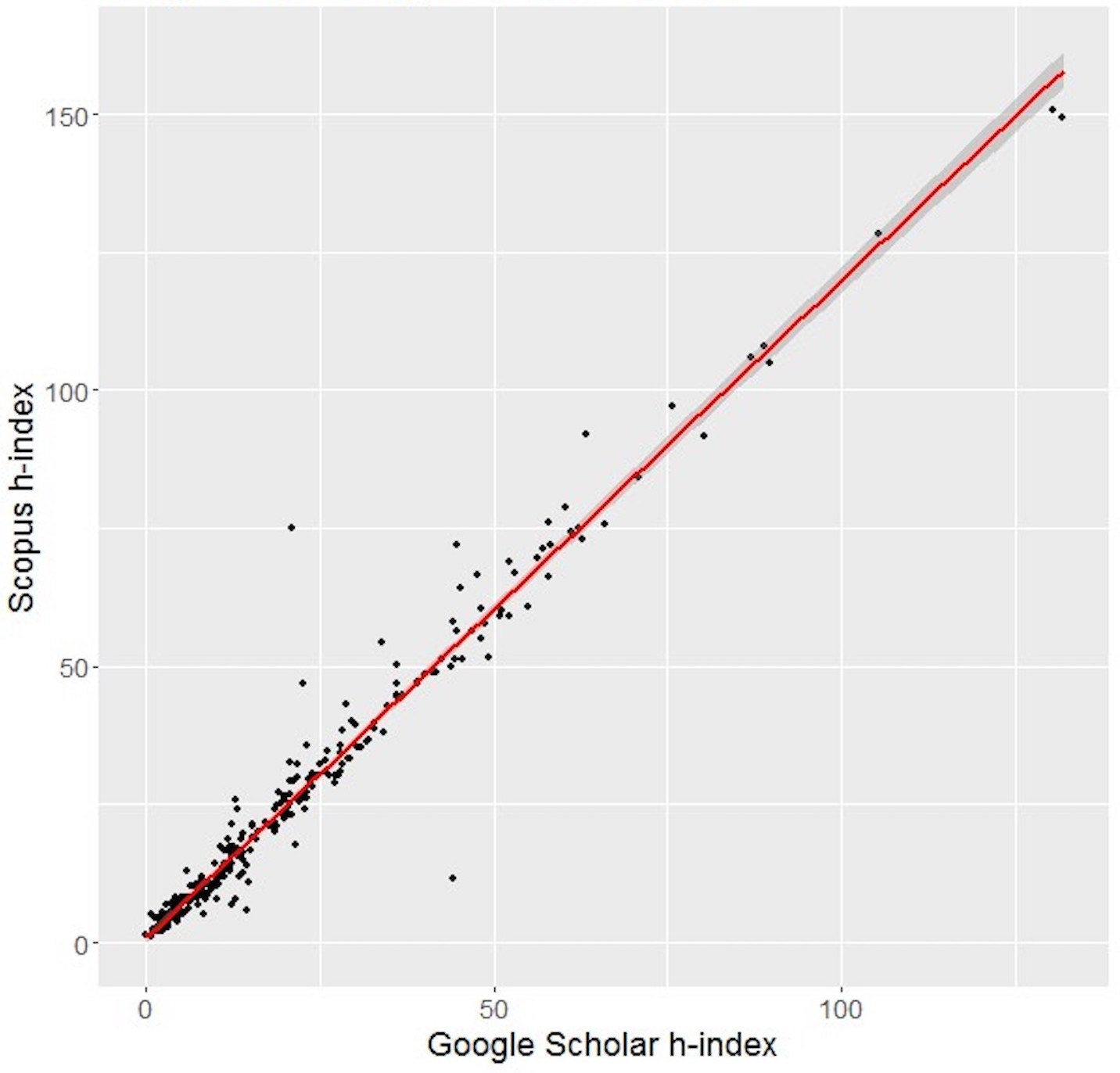

Supplement: Multimedia Appendix 4 [file jmir_v23i5e28859_app4.png]
